# Promoting Women’s Well-Being: A Systematic Review of Protective Factors for Work–Family Conflict

**DOI:** 10.3390/ijerph20216992

**Published:** 2023-10-28

**Authors:** Lucrezia Cavagnis, Claudia Russo, Francesca Danioni, Daniela Barni

**Affiliations:** 1 Department of Human and Social Sciences, University of Bergamo, 24129 Bergamo, Italy; daniela.barni@unibg.it; 2 Experimental and Applied Psychology Laboratory, Department of Human Sciences, European University of Rome, 00163 Rome, Italy; claudia.russo@unier.it; 3 Family Studies and Research University Centre, Catholic University of Milan, 20123 Milan, Italy; francescavittoria.danioni@unicatt.it

**Keywords:** work–family conflict, women, well-being, coping strategies, protective factors

## Abstract

Work–family conflict is a prominent issue, especially in our society, where people are expected to fulfil many roles simultaneously. Work and family life demands significantly impact an individual’s overall well-being, especially for women, since they typically balance caregiving for children and elderly relatives with careers. Therefore, highlighting which factors might protect women from experiencing work–family conflict is essential to enhance women’s and their family’s well-being. Thus, the main aim of the present study was to systematically review previous research on women’s coping strategies and protective factors which can reduce the negative effects of work–family conflict. Following the PRISMA guidelines, we conducted a literature search of three databases (PubMed, PsycINFO, and Scopus). After the screening and the eligibility phases, we included a final set of 13 studies. Most of these studies adopted a cross-sectional design (N = 10), and a few adopted a longitudinal one (N = 3). Results highlighted the role of different personal (e.g., hardiness, self-esteem, locus of control) and relational factors (e.g., family and work support) that significantly reduce the negative effects of work–family conflict in women’s lives. Findings, practical implications, and future research directions are discussed.

## 1. Introduction

In modern society, individuals usually engage in multiple roles simultaneously across different life domains, such as couple, family, work, school, neighbourhood, and community. Successfully managing multiple roles can contribute to enhanced self-esteem, a sense of fulfilment, and personal growth [1,2]. Each role can provide individuals with opportunities for learning, skill development, and new social relations, thereby fostering a sense of purpose and identity integration across different life contexts [3,4]. However, when the demands of multiple roles become overwhelming or conflicting, individuals may experience relevant negative consequences [5].This scenario, referred to as “role conflict”, arises when the expectations and demands associated with different roles clash. Such clashes can lead to stress, guilt, and difficulty in meeting all expectations [6,7]. Research has highlighted that the conflict among some roles might have greater negative effects than others [8]. For example, conflicts between work and family roles have been shown to have more severe negative effects on individuals than conflicts between different work roles [6]. Specifically,work–family conflict is defined as “*a form of inter-role conflict in which the role pressures from the work and family domains are mutually incompatible in some respect. That is, participation in the work (family) role is made more difficult by virtue of participation in the family (work) role*” ([6] p. 77). This inter-role conflict can take two directions [9]. Firstly, the work role can interfere with the family role (WIF), or secondly, the family role can interfere with the work role (FIW). Although negative outcomes may occur in both directions, work–family conflict is more frequent than family–work conflict [10,11] since work boundaries are less permeable than family ones [12].

The effect of work–family conflict should be read in the evolution of social trends that have brought new challenges and responsibilities for individuals in managing their work and family commitments. Social trends (such as the increasing participation of women in the workforce, a greater number of working single-parent and dual-earner families, and the growing caregiving needs of an aging population) are introducing new responsibilities and challenges for both women and men in managing their work and family commitments [13,14]. Scholars have indicated that women generally report higher levels of work–family conflict compared to men [15,16,17]. This is especially significant as the current engagement in multiple roles, including spouse, parent, caregiver, and employee, introduces complex dynamics that impact women’s overall well-being and life satisfaction [18]. Additionally, women are more likely than men to leave their jobs when faced with new caregiving responsibilities [19]. Notably, a considerable percentage of women (65.5%) and a much smaller percentage of men (7.4%) who voluntarily left their jobs in Italy in 2021 attributed their resignations to the challenge of balancing work and caregiving roles [20] (accessed on 14 August 2023). In order to better understand the significant impact of work–family conflict, it is essential to examine its negative consequences on various aspects of individuals’ lives. Additionally, protective factors and coping strategies that can promote women’s wellbeing also need to be addressed.

Extensive research has explored the negative consequences of work–family conflict on various dimensions of well-being and mental health. Indeed, scholars have highlighted an increase in stress levels, burnout, depression, and a decrease in both life and job satisfaction [21,22]. The consequences of work–family conflict on various aspects of individuals’ lives, encompassing private, relational, working, and social domains, can be classified into three distinct groups: work-related outcomes, family-related outcomes, and domain-unspecific outcomes [11]. However, it is of utmost importance to notice that there is a paucity of research focusing on the protective factors and coping strategies related to work–family conflict. Protective factors could act as buffers, shielding individuals from the negative effects of such conflicts, while coping strategies may provide individuals with the resources needed to navigate these challenges effectively. Moreover, this research can inform the development of targeted interventions and policies that foster a more supportive work and family environment. By exploring the mechanisms that could reduce the consequences of work–family conflict, it is possible to promote a healthier work–life balance.

Based on the above evidence, this review first introduces how work–family conflict impacts different life domains. Following that, it provides a systematic summary of the main findings on the topic, focusing on the protective factors and coping strategies that lead women to face the consequences of work–family conflict effectively.

### 1.1. Work-Related Outcomes

In the growing body of research about work–family conflict, work-related outcomes have emerged as the most studied variables, especially in the field of work and organizational psychology. Therefore, work-related aspects are in the foreground of interest [23]. Different work-related outcomes such as job satisfaction, organizational commitment, intention to quit, burnout, absenteeism, work-related strain, and organizational citizenship behaviour have been found to be closely linked to work–family conflict [24,25]. The findings from cross-sectional studies have consistently shown that individuals who experience high levels of work–family conflict are more likely to report lower levels of job satisfaction and organisational commitment [26,27,28,29,30]. Indeed, the competing demands of both work and family domains often lead to increased stress and reduced engagement with one’s job. Furthermore, employees facing significant conflict between their work and family roles are more likely to consider leaving their current position [23]. The persistent effort required to fill responsibilities in both domains (i.e., work and family) can potentially lead to emotional exhaustion. Simultaneously, the inability to fully engage in either domain might foster feelings of depersonalization [31,32]. Additionally, the intricate task of balancing competing priorities from work and family can intensify psychological distress and emotional exhaustion, increasing the likelihood of absenteeism and work-related strain among employees [24].

When considering the negative consequences of work–family conflict, it is fundamental to recognize that organizations’ policies can mitigate its impact. For instance, scholars have highlighted that companies prioritizing work–family balance through the implementation of flexible work arrangements, parental leave policies, childcare facilities, and employee assistance programs can contribute significantly to managing family and work roles [33,34,35]. A study conducted by Medina-Garrido and colleagues [36] highlighted that the existence and accessibility of work–family policies improved employee well-being and, ultimately, job performance. Moreover, they also found that work–family policies directly reduce the intention to leave the organization [37]. These proactive measures can foster an environment where employees can manage their work and family responsibilities more effectively, ultimately resulting in improved well-being and job performance.

### 1.2. Family-Related Outcomes

The second life domain in which work–family conflict has a relevant influence is within the family context and dynamics. Specifically, the most noteworthy family-related outcomes are marital satisfaction, family satisfaction, and family-related strain [23,38], which are all negatively affected by high levels of work–family conflict [39,40,41]. Indeed, when people are torn between work and family responsibilities, their ability to fully engage and participate in family life can be compromised. In this regard, Vieira and colleagues [42] found that parents experiencing work–family conflict were prone to higher levels of irritability in their parenting role, leading to higher levels of internalizing and externalizing symptoms in their children.

Additionally, scholars have reported that the spillover effect (i.e., the generalization of behaviour, emotions, attitudes, or stress of one life domain to another) of work-related stress into the family domain can contribute to elevated levels of family-related strain. Such strain often manifests as reduced patience, irritability, and challenges in fully participating in family activities. This spillover effect creates a ripple through the family environment, affecting its overall dynamics and atmosphere [43,44]. An example of a spillover effect from work to family occurs when, following an employee’s day at work where a supervisor incessantly criticized every error, one’s mind continues to dwell on these mistakes and perhaps the supervisor’s lack of respect. This, in turn, can trigger conflicts with one’s relatives [44].

It is also relevant to consider that work–family conflict does not always affect family dynamics in the same way, as there are several individual [33,45] and family [22,46,47] characteristics that can play a moderating or mediating role. Indeed, Aazami et al. [33] found that maladaptive coping strategies worsened the negative effect of work–family conflict. In contrast, Ho and colleagues [45] found that a higher level of family orientation played a protective role. Moreover, family support is considered a resource that yields positive outcomes (e.g., great energy and positive affect), further enhancing individuals’ workplace performance [22,46]. Additionally, dyadic coping, or the way partners support each other in coping with stress, can play a significant role in balancing work and family. Also, Fallahchai and colleagues [47] highlighted that practical strategies, such as dedicated family time, promoting open communication, and seeking external support when needed, can empower families to navigate work–family conflict more effectively and maintain healthier dynamics.

### 1.3. Domain-Unspecific Outcomes

The third category of factors associated with work–family conflict is labeled as domain-unspecific. Several studies have examined these outcomes, such as life satisfaction, psychological strain, somatic complaints, depression, and substance use or misuse. In this vein, a comprehensive review conducted by Allen and colleagues [21] found that work–family conflict has widespread and serious consequences on individuals and could lead to harmful habits. For example, both Miller et al. [48] and Aazami et al. [33] highlighted that substance use or misuse could be a coping mechanism for dealing with these conflicting responsibilities. Specifically, stress can trigger strong emotional responses that can be challenging to manage. Consequently, some individuals may rely on using substances as a means of coping with these intensified emotions [48]. Additionally, Shin and Jeong [49] investigated work–family conflict as a predictor of depression and work engagement. They found that work–family conflict negatively influenced work engagement and positively influenced depression, indicating that the strain caused by work–family conflict can lead to decreased work engagement and increased depressive symptoms. Furthermore, insomnia is often associated with conflicting responsibilities. Indeed, Wagner et al. [50] suggested that, when stress from work is carried over into the home environment, people feel emotional exhausted, and experience disrupted sleep patterns.

The persistent effort required to manage these conflicting roles can contribute to psychological strain, resulting in a decline in mental health and well-being [51]. Moreover, the stressors associated with balancing the demands of work and family domains can manifest as physical symptoms such as headaches, fatigue, and muscle tension. Indeed, individuals experiencing high levels of work–family conflict appeared to be more prone to suffer from somatic complaints [52].

However, some people may develop coping strategies to effectively handle conflicts, which can result in improved well-being. For example, Hall [53] studied the protective effect towards work–family conflict of taking initiative (adaptively tuning one’s behaviour to handle work–family conflict actively), seeking help (requests for help from family members or colleagues), and redefinition (adjusting the standards for self-evaluation).

Furthermore, individuals can actively enhance their ability to handle work–family conflict by implementing various strategies, for example, building and nurturing social support networks, balancing priorities, seeking support from mental health professionals, implementing time management, improving assertive communication, and accessing employee assistance programs [53,54,55].

Moreover, different studies pointed out that the relationship between work and family domains is influenced by several mediating and moderating factors (e.g., social support, personality traits, and coping strategies), which can mitigate the negative effects of work–family conflict on well-being [54,55]. For instance, family orientation, conceptualized as a relationship-oriented personality trait, showed a negative association with work–family conflict. Those with a strong family orientation tend to seek ways to balance their work and family responsibilities proactively. They make strong efforts to allocate their time and resources effectively, trying to maintain a harmonious integration of their roles. Moreover, social support has been identified as an important moderator of stress, reducing the negative effects of work–family conflict on well-being. Indeed, when individuals receive substantial support from their family members, friends, or colleagues, they often experience a significant reduction in stress levels. This support can manifest in various forms, including emotional support, instrumental assistance, or even understanding and encouragement. Strong social support allows individuals with the resources necessary to effectively cope with the challenges posed by work–family conflict [56].

### 1.4. The Present Study

The intricate interplay between work and family domains has undergone significant transformation in recent decades, supported by changing social norms, evolving workforce dynamics, and an increasing awareness of the centrality that family well-being holds in the broader context of public health. In this landscape, the concept of work–family conflict has emerged as a salient issue, impacting both individuals and the community. Research on work–family conflict has mainly focused on its negative consequences, whereas protective factors and coping strategies have not been extensively addressed. Nonetheless, the study of such factors is paramount to enhance family well-being. Understanding these factors may, indeed, fill the research gap and can yield significant benefits for individuals, families, employers, and policymakers. By identifying coping strategies and protective factors, individuals can better manage their work and family responsibilities, reducing stress and enhancing their quality of life. Families can benefit from improved communication, and shared responsibilities, fostering healthier family dynamics. Employers could implement supportive workplace policies such as flexible work arrangements, and childcare assistance. Moreover, policymakers could promote policies that support working women and their families. For example, parental leave, affordable childcare, and flexible work hours can be proposed.

Additionally, these results can contribute significantly to public health and social change. Firstly, reducing stress and improving well-being could lead to improved mental health outcomes for individuals (e.g., lower anxiety, and irritability). Secondly, as families benefit from improved communication and healthier dynamics, there can be positive effects on family relationships and children’s well-being. This, in turn, can have long-term implications for child development, and family cohesion. Furthermore, a supportive workplace can, consequently, also contribute to increased productivity and decreased absenteeism. These factors can significantly impact economic and societal well-being. In addition, policymakers could address work–family conflict at a systemic level, benefiting both individuals and society.

Building upon the current state of the art, this systematic review aims to comprehensively summarize and discuss the existing body of literature concerning women’s coping strategies and protective factors for work–family conflict. While it is essential to recognize that both men and women experience work–family conflict, we decided to focus specifically on women due to their primary responsibility for household tasks while simultaneously pursuing significant careers [36]. Indeed, this situation often leads to higher levels of work–family conflict for women compared to men [18,57]. By examining protective factors and coping strategies, we hope to shed light on effective strategies that contribute to enhancing the well-being of women and their families.

## 2. Materials and Methods

In conducting the present systematic review, the PRISMA (Preferred Reporting Items for Systematic Reviews and Meta-Analyses) statements were followed, and compliance with these guidelines has been ensured by completing the PRISMA checklist [58], available in the Supplementary Materials. PRISMA is a set of guidelines designed to ensure the comprehensive reporting of systematic reviews and meta-analyses. By following PRISMA, we aimed to provide a clear and structured research process, including study selection, data extraction, and synthesis of findings.

This systematic review was preregistered in the OSF database (https://osf.io/yqxer/?view_only=5c329202152e4e63af9fc256691e1068, accessed on 20 September 2023).

A systematic narrative approach to report the studies’ main findings was adopted. Indeed, the high heterogeneity in the studies related to the methods, variables (e.g., family, relationship, and job satisfaction, behavioral coping strategies, personality traits, and social support), and the lack of detailed statistical information in many studies precluded the possibility of conducting a meta-analysis.

Furthermore, considering the paucity of studies available, providing scholars and practitioners with a qualitative description of the key findings rather than a quantitative one might be more informative for the development of future lines of research and intervention.

### 2.1. Search, Screening, and Selection Strategies

**Figure 1:**
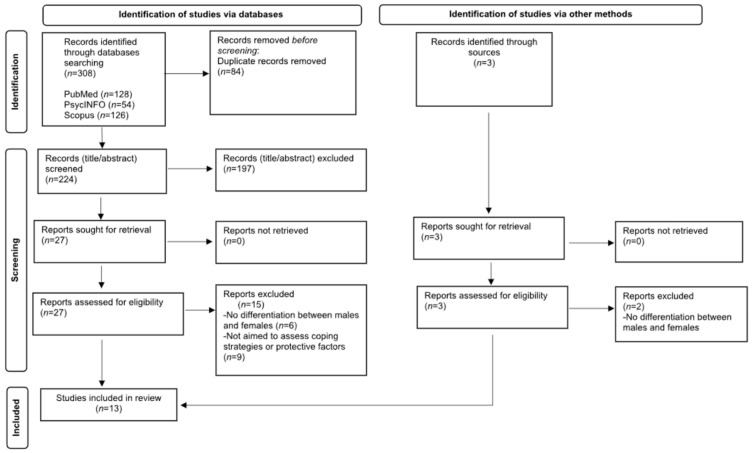
Flowchart for the systematic review procedure. Adapted from Liberati et al. [60].

The first search was conducted in May 2023, with the last update completed on 10 July 2023. We were interested in research concerning the protective factors and/or coping strategies for work–family conflict among women. The studies were identified relying on the following databases: PubMed, PsycINFO, and Scopus. We decided to consider these databases because they are widely recognized as the most comprehensive and authoritative sources for psychological research [59]. We adopted an iterative search strategy with the combination of the following terms: (“work–home conflict” OR “work–family conflict”) AND (“protective factor*” OR “coping” OR “well-being*”) AND (“women” OR “female*”). We did not apply any limits or filters. In Figure 1 we summarize the steps of the search strategy.

The searches and screening were run and managed on EndNote X8 [61].

A total of 308 studies were initially matched. During the identification phase, we adopted the following inclusion criteria: (1) peer review research articles written in English or Italian based on the language competencies; (2) research articles aimed at investigating the protective factors or coping strategies in the work–family conflict; (3) research articles with cross-sectional or longitudinal design. We excluded: (a) books, book chapters, and conference proceedings. These sources were excluded to focus on peer-reviewed research articles, which typically undergo a review process before publication; (b) grey literature such as reports, theses, dissertations, and working paper. We excluded grey literature to prioritize the inclusion of studies with a higher level of peer review and quality assurance; (c) research articles concerning the negative effects of work–family conflict on well-being since our specific research focus was on identifying protective factors and coping strategies related to work–family conflict among women.

After removing duplicates (N = 84), 224 studies were screened for title and abstract. During the screening phase, if an abstract was not available or it was not informative enough to determine its inclusion or exclusion, we proceeded with the full-text screening. For example, some abstracts provided only brief descriptions of the study objectives without specifying the participant demographics or outcomes of interest. In such cases, we proceeded with the full-text screening to gain a more comprehensive understanding of the study’s relevance to our research question. After this phase, 197 records were excluded, and the remaining 27 were further examined by reading the full texts. During the full screening process, the following eligibility criteria were adopted: (a) articles aimed at investigating factors that can enhance women’s well-being or that can counteract the negative effect of work–family conflict; (b) articles that took into account mediators and moderators between work–family conflict and well-being; (c) research articles with a clear distinction between males and females in the results section.

### 2.2. Coding

The selected studies were read in their full text. Following that, a coding scheme was employed. For each article, the authors, year, country, study design, sample size, participants characteristics, and fundings were reported in Table 1. Further, concerning the association between the protective factors and participants’ wellbeing, the result section of each article was read in detail, and the statistical estimates were reported in the results section of the article. Additionally, to ensure the overall quality of the included studies, we completed the AXIS checklist.

### 2.3. Quality Assessment

We employed the appraisal AXIS tool for observational and cross-sectional studies. The AXIS is a critical appraisal tool designed to assess the overall quality of the study, reporting, and the risk of bias [69]. It is composed of a checklist of 20 items that evaluate different aspects of study quality, including study design, sample size justification, sample representativeness, measurement validity and reliability, description of statistical methods, and reporting of funding and conflicts of interest. Items were scored as follows: Yes = 1, No = 0, and Don’t know = 0. A total quality score from 0 to 20 was assigned to each study, with higher scores indicating a higher assessed quality. When assessing the quality scores using the AXIS tool, our approach aligns with the classification used in previous literature reviews e.g., [70,71]. This classification involves three levels: low quality (0–7 points), moderate quality (8–14 points), and high quality (15–20 points). For the longitudinal studies, our methodology follows the precedent set by previous reviews [72]. In such cases, we focus exclusively on the data from the initial wave when responding to the AXIS items.

## 3. Results

### 3.1. Main Characteristics of the Selected Studies

Overall, 13 studies were included in the systematic review (see Figure 1). An Excel spreadsheet was prepared to summarize the relevant information from the selected studies (i.e., authors, year of publication, country, study design, sample characteristics, and fundings). All the included studies were independently and simultaneously coded used the coding scheme (Section 2.2) by two raters with experience in the topic under investigation (the agreement percentage was 95%) with reference to the above information. Discrepancies were discussed with a third rater and resolved among the three evaluators. A summary of the characteristics of the included studies is presented in Table 1.

In terms of year of publication, the studies were published between 2007 and 2023. They were conducted in Malaysia (N = 2), the Balearic Islands (N = 1), the United Kingdom (N = 1), China (N = 2), Portugal (N = 2), Spain (N = 1), Israel (N = 2), and the remaining two studies presented participants from different countries, such as Australia, New Zealand, China, and Hong Kong, and Austria, Belgium, Finland, Germany, the Netherlands, Portugal, and Switzerland, as they were part of a larger research project. The total number of participants was 7983 (M = 614.08, SD = 620.28). Most samples (N = 8) were gender-balanced, whereas five studies were conducted with only women. Most studies (N = 7) reported one funding source. In terms of research design, almost all the studies adopted a cross-sectional design (N = 10), while the remaining three studies used a longitudinal design. All the studies employed a quantitative approach and utilized self-report measures (i.e., questionnaires), with the only exception being the research by Somech and Drach-Zahavy [67], in which they used questionnaires in conjunction with interviews.

While reading the full texts of the records collected, we identified, through an inductive thematic approach, two main categories: individual protective factors and relational protective factors. The results of the selected records were then interpreted according to these categories and are reported below.

### 3.2. Individual Protective Factors

Individual protective factors encompass personal attributes, characteristics, and strategies that contribute to an individual’s ability to manage and mitigate the challenges posed by work–family conflict effectively. These factors operate at an intrapersonal level and play a central role in promoting well-being and reducing the negative impact of work–family conflict. Five studies examined these factors, showing heterogeneous results. Aazami and colleagues [33] investigated the role of individuals’ behavioural coping strategies, both adaptive (i.e., active coping, positive reframing, acceptance, planning, use of instrumental and emotional support, religion, self-distraction, venting, and humour) and maladaptive (i.e., self-blame, substance use, denial, and behavioural disengagement) in the association between work–family conflict and psychological distress among women. The study found that maladaptive coping strategies played a significant mediating role in work–family conflict (*b* = 0.33, *p* < 0.001), worsening psychological distress. On the other side, adaptive coping strategies did not significantly mitigate this relationship (*b* = −0.02, *p* = 0.65).

Ho and colleagues [45], with dual-earner couples, examined the roles of a personality trait (i.e., family orientation) and perceived social support (from family and work) as predictors of work–family conflict. Results showed a negative association between work–family conflict and family orientation. Specifically, women with higher levels of family orientation reported significantly lower levels of work–family conflict (*β = −*0.43, *p* < 0.001). On the other hand, support from family and work (supervisor and colleagues) was not associated with reduced work–family conflict (*β =* 0.02 and *β = −*0.04, respectively).

Recuero et al. [32] investigated how behavioural (i.e., rational strategies oriented to solve the problem or control the situation) and emotional (i.e., emotional strategies oriented to tolerate the situation) coping moderated the relationship between work–family conflict and two dependent variables: emotional exhaustion and depersonalization. First, they found that, compared to women with high behavioural coping strategies, women with low behavioural coping showed more emotional exhaustion in situations of low conflict, but less emotional exhaustion in situations of high conflict. Second, they also found that women who employed higher emotional coping strategies experienced less emotional exhaustion compared to women who employed fewer emotional coping strategies when facing low work–family conflict. Third, compared with women using fewer behavioural coping strategies, women using higher behavioural coping strategies showed: (i) lower levels of depersonalization in low work–family conflict; (ii) higher level of depersonalization in high work–family conflict.

The study conducted by Somech and Drach-Zahavy [67] examined the relationships between sex, gender role ideology (traditional vs. non-traditional), several coping strategies, and work–family conflict. Through the interviews, they identified different coping strategies such as good enough at home, super at home, delegation at home, priorities at home, good enough at work, super at work, delegation at work, and priorities at work. Findings highlighted an interaction between sex, gender role ideology, and coping strategies on work–family conflict. The main results revolved around three coping strategies: being good enough at home, being good enough at work, and delegation at work. The first coping strategy implied a lowered performance of family responsibilities to a less-than-perfect level, the second implied a reduced work performance to a less-than-perfect level, while the third was based on delegating one own’s work to others. The analyses showed that, for low scores on the “good enough at home” coping strategy, traditional women scored lower on work–family conflict than nontraditional women, whereas for high scores on this strategy, traditional women scored higher than nontraditional women. Furthermore, this interaction pattern was inverted when looking at the “good enough at work” and “delegation at work” coping strategies. Here, traditional women scored higher on work–family conflict than nontraditional women in low usage of such coping strategies, but for high usage of these coping strategies nontraditional women scored higher of work–family conflict than traditional women. When focusing only on traditional women, they showed less work–family conflict when employing the “good enough at work” coping strategy *t* = −2.17, *p* < 0.05).

Matias and Fontaine [57] examined different coping strategies in dual-earner couples, such as partner coping, positive attitudes towards multiple roles, management and planning skills, professional adjustments, and institutional support. They found that women’s management and planning skills (i.e., planning, managing time, being flexible, and segmenting work and family) were the only strategies negatively associated with work–family conflict (*β* = −0.27, *p* < 0.05). Surprisingly, and contrary to the expectations, having a positive attitude towards multiple roles revealed a positive relationship with work–family conflict (*β* = 0.32, *p* < 0.01). The other strategies did not show any significant association.

### 3.3. Relational Protective Factors

Relational protective factors include dynamics related to one’s family and work systems. Specifically, family-related protective factors refer to a set of elements within the familial context that contribute to individuals’ ability to effectively manage and mitigate the challenges from work and family domains, promoting well-being. Work-related protective factors encompass elements within the work environment that can help to reduce the negative impacts of work–family conflict and contribute to overall well-being.

Aazami and colleagues [62] conducted a study with a Malaysian sample of women to explore how family and job satisfaction mediated the association between work–family conflict and psychological distress. More specifically, the authors adopted a two-dimensional measure for work–family conflict that assessed both strain-based (i.e., strain and fatigue associated with one role, cross over into another role and affect performance) and time-based conflict (i.e., time spent to fulfil one’s responsibilities in one domain limits the amount of time that is available to the other domain). The authors, in addition to a direct effect, found that family satisfaction mediated the association between strain-based work–family conflict and psychological distress, supporting the idea that family satisfaction might play a protective role. On the contrary, time-based work–family conflict was not associated with well-being.

The study conducted by Andrade and Mikula [63] examined the effect of several mediators, such as distributive and procedural justice household labour, distributive and procedural justice childcare, and relationship satisfaction as protective factors in the relationship between work–family conflict and relationship satisfaction. Results showed that all mediators played a significant role (distributive and procedural justice household labour, *β* = 0.13, *p* < 0.05, *β* = 0.11, *p* < 0.05; distributive and procedural justice childcare, *β* = 0.14, *p* < 0.05, and *β* = 0.12, *p* < 0.05). Specifically, women who felt to be supported by their partners for household work and childcare reported higher levels of both relationship satisfaction and wellbeing.

Drummond et al. [54] conducted a longitudinal and cross-cultural study, with participants from Australia, New Zealand, China, and Hong Kong. They tested a model whereby the effect of support (from both family and work supervisors) at T1 on strain and satisfaction (both in the family and job context) at T2 was mediated by work–family conflict (at T1). Their analyses indicated that, for women, both family (*b* = −0.027, *p* < 0.05) and supervisor support (*b* = −0.038, *p* < 0.01) acted as protective factors toward work–family conflict. When considering the geographic region as a moderator, the authors also found that supervisor support reduced work–family conflict for Chinese and Hong-Kong participants (*b* = −0.052, *p* < 0.01), both men and women. This result is consistent with collectivistic cultures which have strong family-oriented values [73]. Indeed, here, supervisors may be more understanding of family commitments, thus reducing work–family conflict.

The study carried out by Matias and colleagues [66] focused on workplace family support (i.e., supportive behaviours directed toward the performance of the parental role) and its impact on work–family conflict of dual-earner couples. In particular, the authors predicted that workplace family support would act as a protective factor for work–family conflict, and this effect would be both direct and mediated by parental satisfaction. When considering women, the focus of the present review, the results showed that the authors’ hypotheses were partially supported. Indeed, the direct effect of workplace family support on work–family conflict was not statistically significant; on the contrary, its effect on work–family conflict through parental satisfaction was significant (*β* = −0.07), indicating a fully mediated effect of workplace family support on work–family conflict.

Pan et al. [25] examined the relationship between work–family conflict, family support, subjective happiness, and organizational citizenship behaviour (OCB) among a sample of women through a moderated mediation model. They predicted that the relationship between work–family conflict and OCB was mediated by subjective happiness and that the effect of work–family conflict on subjective happiness was moderated by family support. Results showed that subjective happiness played a mediating role in the relationship between work–family conflict and OCB (*β* = 0.41; *p* < 0.001). Additionally, the negative effect of work–family conflict on subjective happiness was smaller for women that had more family support.

Kulik [68] did not directly investigate the effect of protective factors of role conflict; rather, based on the assumption that three coping strategies (taking initiative, help-seeking, and redefinition) are effective for reducing work–family conflict, the author aimed to explore which environmental (i.e., spousal and family support, support from the workplace, and division of household tasks) and personal (i.e., hardiness, gender-role ideology, and wife’s income advantage over her husband) resources were positively associated with the adoption of the above coping strategies. Furthermore, the author conducted a cross-cultural study with both Jewish and Muslim Arab women. The results showed that, for Jewish women, there was a significant association between spousal support (*r* = 0.39, *p* < 0.01), family support (*r* = 0.30, *p* < 0.05), hardiness (*r* = 0.30, *p* < 0.05), the division of household tasks (*r* = 0.28, *p* < 0.05), and the utilization of the help-seeking coping strategy. Additionally, support from the workplace (*r* = 0.31, *p* < 0.001) and an even distribution of household tasks (*r* = 0.23, *p* < 0.05) were significantly linked to the use of the taking initiative coping strategy. Furthermore, for Muslim Arab women, the results highlighted that the redefinition coping strategy was positively correlated with spousal support (*r* = 0.29, *p* < 0.01), and workplace support (*r* = 0.25, *p* < 0.05). In contrast, an inverse relationship emerged between a wife’s income advantage over her husband and the redefinition strategy (*r* = −0.22, *p* < 0.05).

Jewish women, living in modern societies with more flexible gender roles, tended to utilize active coping strategies. In contrast, Muslim Arab women, who live in traditional societies may avoid using available resources due to cultural constraints on women’s status.

The study carried out by Dutta et al. [65] explored the outcomes of a mentoring program for female academics through a longitudinal study. It sought to explore through which mechanisms mentoring facilitates the professional development of female academics. The authors developed a pilot mentoring program and evaluated various health-related and attitudinal measures (i.e., self-esteem, self-efficacy, job-related wellbeing, work–family conflict, and job satisfaction) at baseline, six months, and one year into the mentoring relationship. The results showed that mentoring had positive effects on both mentees and mentors. Specifically, over the course of one year, mentees experienced improvements in job-related well-being (anxiety-contentment, *p* < 0.05), self-esteem, *p* < 0.001, self-efficacy, *p* < 0.05, and a reduction in work–family conflict, *p* < 0.05).

Chela-Alvarez and colleagues [64] conducted a cross-sectional study to investigate both individual and relational (risk and protective) factors from experiencing work–family conflict with a sample of female hotel housekeepers in the Balearic Islands. The findings yielded several noteworthy outcomes. For example, low social support and an external locus of control were associated with difficulties in work–life balance, whereas job and wage satisfaction (OR = 0.94, *p* < 0.005; OR = 0.96, *p* < 0.05) emerged as protective factors of work–family conflict, serving to mitigate the challenges posed by work and family domains.

### 3.4. Quality Assessment

The average quality score of the included studies was 16.62 out of a total of 20 points (min = 14, max = 14, SD = 1.12). In accordance with the AXIS tool [69], this suggests that the studies were of high quality, as shown in Table 2.

## 4. Discussion

In recent decades, the role of women in modern society has significantly changed due to their increasing participation in the labour market. Despite the increasing number of dual-earner families, there is still not an egalitarian division of tasks in the family, with women providing most of the care to their children and elderly parents [74]. Consequently, balancing work and family demands appears to be a relevant issue, particularly for women. Indeed, they are exposed to a higher risk of experiencing negative consequences arising from the competing demands of work and family [18,57].

To understand how to enhance women’s well-being, it is crucial to consider their coping strategies and protective factors that can prevent them from experiencing the negative consequences of work–family conflict.

For these reasons, the current systematic review aimed at extending prior knowledge on this topic by summarizing and discussing the research articles focused on women’s coping strategies and other protective factors associated with work–family conflict.

Overall, most studies investigated individual and relational factors through a cross-sectional design, while a small portion of the included studies employed a longitudinal design. Findings revealed heterogeneous evidence. For example, Matias and Fontaine [57] found that behavioural coping strategies counteracted the negative effect of work–family conflict on women’s well-being. On the contrary, Recuero and Segovia [32] found that neither behavioural nor emotional coping strategies were significantly associated with women’s work–family conflict. However, the authors took a step forward and found that employing behavioural coping strategies was effective in reducing women’s emotional exhaustion only when the level of conflict was low. According to the authors, this could be explained considering that problem-focused coping strategies can be effective only when women have agency in the control of the potential stressors [75,76]. A similar interpretation was provided by Aazami and colleagues [33] who operationalised behavioural coping strategies as either adaptive or maladaptive. Contrary to their expectations, adaptive coping strategies exhibited no statistically significant association with work–family conflict.

Moreover, maladaptive coping strategies showed a positive correlation with work–family conflict indicating that an increased reliance on such strategies heightened women’s conflict levels. As suggested by Recuero and Segovia [32], a lack of control in stressful situations, such as work–family conflict, can trigger women’s negative effects of behavioural coping.

Importantly, the effectiveness of coping strategies is not solely determined by the level of control over work and family demands; individual factors also exhibit a significant influence. In this regard, Somech and Drach-Zahavy [67] found that attitudes towards gender roles (e.g., traditional vs. nontraditional) had a significant impact. Indeed, they demonstrated that, for traditional women, employing the “good enough at work” strategy reduced work–family conflict, whereas the “good enough at home” strategy was counterproductive. On the contrary, nontraditional women showed an opposite pattern. This result is interesting as it highlights how important personal values and beliefs are in the context of work–family conflict. Values and beliefs shape the perception of ourselves, others, and the word around us, guiding our attitudes and actions [77]. Personal values represent a core aspect of personal identity as much as people tend to depict themselves in terms of their value priorities [78]. Thus, people who live and act according to their values are more likely to feel a sense of authenticity [79,80]. Indeed, for traditional women who perceive their primary life roles as mothers and wives, any coping strategy that enables them to fulfil these expectations and maintain high standards at home could function as an effective approach to reduce work–family conflict. By lowering their work responsibilities (such as using the “good enough at work” and “delegation at work” strategies), these women can balance work obligations while prioritizing family obligations in line with their gender-role ideology, and consistently with their self-concept, leading them to experience less work–family conflict. On the other hand, nontraditional women might evaluate their self-worth based on their work accomplishments [81], so they tend to establish elevated standards for their performance in the workplace, which can explain the results by Somech and Drach-Zahavy [67] whereby when nontraditional women employing the “good enough at work” strategy, they suffered more work–family conflict.

Interestingly, a relationship-oriented personality trait emerged as an important protective factor for work–family conflict. Specifically, in the study conducted by Ho and colleagues [45], family orientation, which encompasses the extent to which individuals have a strong sense of family solidarity and maintain a loving relationship with their family members, was negatively associated with work–family conflict. Therefore, strategies employed to manage work–family conflict are not solely derived from women’s beliefs and value systems. Rather, intrinsic aspects of an individual’s personality also play a significant role. Despite this, since personality traits are generally considered to be relatively stable across lifespan [82,83], interventions aimed at dealing with work–family conflict might be more effective if they target aspects of women that are more adaptable.

When considering the more adaptable and flexible factors related to women’s lives, it becomes essential to consider their interpersonal relationships. Indeed, the quality of family relationships (with the partner, children, and other family members) and work relationships (e.g., with other colleagues) could facilitate or hinder the implementation of effective coping strategies, even on an individual level. It is entirely plausible that the quality of family relationships and the quality of work relationships are interdependent, but their effects on work–family conflict might have a different weight. For example, Higgins et al. [84] found that work conflict was more important than family conflict in predicting work–family conflict. This would be due to the fact that people have less control over their work lives than their families. Adopting an ecological systems perspective could help in better understanding these mesosystemic mechanisms and developing psychosocial actions to reduce work–family conflict or promote work–family enhancement.

In this context, Kulik [68] posited that coping strategies not only predict work–family conflict, but a range of environmental factors shape them. According to this perspective, coping strategies mediate the connection between environmental factors and work–family conflict. Indeed, relational aspects such as support from spouses, family members, and an equitable distribution of household responsibilities promote the adoption of help-seeking coping strategy, which, in turn, is expected to reduce work–family conflict. Similarly, support from the workplace also encourages the use of effective coping mechanisms. While these findings might appear intuitive, they hold significant implications as they reveal that women are less likely to seek assistance when they perceive a lack of support from their significant others or workplace. Consequently, they might feel overwhelmed by the conflicting demands of their roles. From a practical perspective, the discovery that effective coping strategies can be facilitated carries both positive and negative implications. On the positive side, this suggests the potential for intervention based on an ecological theoretical framework, which becomes particularly relevant since both women’s family and work environments can be seen as interrelated systems. Conversely, there is the concern that women’s families and workplaces might not be cooperative and may resist participating in such interventions.

In essence, intervening only with women experiencing work–family conflict might not be sufficient. However, even in this case, there is still the possibility to reduce psychological distress derived from work and family conflict. Indeed, Aazami and colleagues [62] assumed that job and family satisfaction could mediate the association between work–family conflict and women’s wellbeing. Their study yielded evidence supporting family satisfaction’s role as a mediator.

Similarly, Andrade’s and Mikula’s [63] findings suggest a similar line of reasoning. They found that factors such as distributive and procedural justice in household labour, distributive and procedural justice in childcare, and relationship satisfaction mediated the association between work–family conflict and women’s wellbeing. Based on this evidence, a straightforward intervention as simple as a weekly plan of both women’s and men’s tasks for household and childcare responsibilities might have a triple positive impact. Firstly, it could alleviate women’s responsibilities concerning home and childcare tasks. Secondly, it could enhance women’s satisfaction with their family and spouse. Thirdly, the improved satisfaction could, in turn, contribute to enhanced well-being for women.

The importance of these considerations is amplified when taking into account the study conducted by Drummond et al. [54]. Their findings emphasized that women’s satisfaction with their relationships has a lasting impact. Indeed, the authors found that support from family and work supervisors in the first wave was negatively associated with participant’s work–family conflict in the second wave. This insight underlines that family and supervisor support serve as enduring protective factors against work–family conflict. Such support also needs to be “instrumental”, with a redistribution of childcare and housework responsibilities.

Similarly, Matias et al. [66] and Dutta and colleagues [65] highlighted that supportive workplace environments could improve women’s well-being and, therefore, reduce work–family conflict.

### Limitations and Future Directions

The findings from this systematic review should be considered considering some limitations. First, our inclusion criteria focused solely on research articles published in English or Italian. Therefore, we did not account for potentially pertinent articles published in other languages. Additionally, we excluded books, book chapters, conference proceedings, and grey literature due to their non-peer-reviewed nature. Despite several concerns regarding including grey literature, often insignificant statistical models are unpublished. Therefore, our conclusions might be affected by the publication bias. Future research should integrate both peer-reviewed and non-peer-reviewed sources, prioritising interpreting the results attentively.

Secondly, the included studies exhibited a great heterogeneity concerning the variables (i.e., independent, dependent, mediators, and moderators). Consequently, this variety prevented us from conducting a meta-analysis, which could have yielded a more precise estimate of the impact of coping strategies or protective factors in managing work–family conflict.

Furthermore, a noteworthy limitation pertains to the nature of the included studies, as they all relied on self-reported measures, which can be influenced by response bias. Only one study [67] employed a mixed-method approach (interviews and questionnaires) to investigate women’s strategies for dealing with work–family conflict. They subsequently tested the effectiveness of the identified strategies using quantitative methods. It would be important for future studies to employ both methods. For instance, analysing the interview transcripts through a qualitative approach, such as a thematic analysis, could provide new insights to deepen our understanding of how to balance work and family responsibilities successfully.

Moreover, most studies used a cross-sectional design, limiting the ability to establish causal relationships. Future longitudinal studies are needed to comprehensively assess the effectiveness of various coping strategies or protective factors over time.

Additionally, sample size calculations were not provided for the included studies. Hence, research articles might have relied on non-representative samples, potentially impacting the statistical significance of their results. Therefore, future research should incorporate an a priori sample size calculation to ensure the use of representative samples and, in turn, give more strength to the results.

## 5. Conclusions

This systematic review provided a synthesis of cross-sectional studies and a limited number of longitudinal research on women’s protective factors and coping strategies to deal with work–family conflict. While previous research has examined such aspects, this review offers a comprehensive summary, providing a more holistic understanding of the subject.

The primary added value of this manuscript lies in its potential to enhance women’s well-being when facing work–family conflict.

Our findings highlight the importance of adopting an ecological perspective to address this significant issue. This implies acting on several fronts. Firstly, empowerment interventions can be developed and implemented to strengthen the protective impact of individual factors in women’s lives, including personality traits, self-esteem, locus of control, and hardiness. These interventions can equip women with the skills needed to deal with their different roles and responsibilities effectively.

However, we acknowledge that solely focusing on enhancing individual factors might prove inadequate. Consequently, it becomes crucial to establish conditions that promote effective coping strategies, thereby counteracting the negative consequences of work–family conflict. These essential conditions extend beyond the individual and encompass the broader contexts in which women operate. Our review highlights the significance of nurturing supportive relationships within women’s close networks, including family members, as well as their workplace and colleagues. These relationships serve as essential conditions that empower women to employ coping strategies effectively. By fostering understanding, collaboration, and empathy in both family and workplace environments, women can better balance work and family responsibilities.

Intervention programs could be proposed with a comprehensive understanding of all the factors at play. Additionally, such programs should be designed for individuals and the contexts (e.g., family and workplace) they are a part of. By adopting this approach, an ecological intervention can be implemented, enhancing women’s well-being and ultimately leading to a more harmonious balance between work and family responsibilities.

## Figures and Tables

**Table 1:** Characteristics of studies included in the systematic review.

Author(s)	Year	Country	Study Design	Sample Size	Participants Characteristics	Fundings
Aazami, S., Akmal, S., & Shamsuddin, K. [62]	2015	Malaysia	Cross-sectional	567 women	M_age_ = 33.4 M_number of children_ = 1.7 M_work hour_ = 42.9	No
Aazami, S., Shamsuddin, K., & Akmal, S. [33]	2015	Malaysia	Cross-sectional	429 women	M_age_ = 34.7 M_number of children_ = 1.7 M_work hour_ = 42.7	Yes
Andrade, C. & Mikula, G. [63]	2014	Austria Belgium Finland Germany Netherlands Portugal Switzerland	Cross-sectional	1512 women	M_age_ = 35 M_number of children_ = 1.8 M_work hour_ = 33	Yes
Chela-Alvarez, X., Garcia-Buades, M.E., Ferrez-Pereira, V.A., Bulilete, O., & Llobera, J. [64]	2023	Balearic Islands	Cross-sectional	1043 women	M_age_ = 43.3 M_work hour_ = 47	Yes
Drummond, S., O’Driscoll, M.P., Brough, P., Kalliath, T., Siu, O., Timms, C., Riley, D., Sit, C., & Lo, D. [54]	2017	Australia New Zealand China Hong Kong	Longitudinal (Two waves with a 12 month time lag)	2183 total sample 1654 women	Total sample M_age_ = 36.33	Yes
Dutta, R., Hawkes, S.L., Kuipers, E., Guest, D., Fear, N.T., & Iversen, A.C. [65]	2011	United Kingdom	Longitudinal (Three waves with a 6 month time lag)	90 total sample 44 women	Not reported	No
Ho, M.Y., Chen, X., Cheung, F.M., Liu, H., & Worthington, E.L. [45]	2013	China	Cross-sectional	306 total sample 153 women	Men M_age_ = 34.4 Women M_age_ = 32 Men M_work hour_ = 48.7 Women M_work hour_ = 44.7	No
Matias, M., & Fontaine, A.M. [57]	2015	Portugal	Cross-sectional	200 total sample 100 women	Total sample M_age_ = 36 Men M_work hour_ = 61 Women M_work hour_ = 57	Yes
Matias, M., Ferreira, T., Vieira, J., Cadima, J., Leal, L., & Mena Matos, P. [66]	2017	Portugal	Longitudinal (Two waves with a 12 month time lag)	180 total sample 90 women	Total sample Age range = 23 to 50 Total sample M_work hour_ = 35	Yes
Pan, Y., Aisihaer, A., Li, Q., Jiao, Y., & Ren, S. [25]	2022	China	Cross-sectional	386 women	M_age_ = 31.39	Yes
Recuero, L.H., & Segovia, A. [32]	2021	Spain	Cross-sectional	262 total sample 161 women	Total sample M_age_ = 38.4	No
Somech, A., & Drach-Zahavy, A. [67]	2007	Israel	Cross-sectional	679 total sample 400 women	Total sample M_age_ = 37	No
Kulik, L. [68]	2012	Israel	Cross-sectional	146 women (59 Jewish, 87 Muslim Arab)	Jevish M_age_ = 36.5 Muslim Arab M_age_ = 35.2 Jevish M_number of children_ = 2.6 Muslim Arab M_number of children_ = 3.3	No

**Table 2:** Quality Assessment and total scores using the appraisal tool for cross-sectional studies (AXIS).

Author(s) and Year	Q1	Q2	Q3	Q4	Q5	Q6	Q7	Q8	Q9	Q10	Q11	Q12	Q13 *	Q14	Q15	Q16	Q17	Q18	Q19 *	Q20	Total Quality Score/20	Quality Rating
Aazami et al. [62]	Y	Y	Y	Y	Y	Y	N	Y	Y	Y	Y	Y	N	N	Y	Y	Y	Y	N	Y	18	High
Aazami et al. [33]	Y	Y	Y	Y	Y	Y	N	Y	Y	Y	Y	Y	N	N	Y	Y	Y	Y	Y	Y	17	High
Andrade and Mikula [63]	Y	Y	Y	Y	Y	Y	N	Y	Y	Y	Y	Y	N	N	Y	Y	Y	Y	Y	NR	16	High
Chela-Alvarez et al. [64]	Y	Y	Y	Y	Y	Y	N	Y	Y	Y	Y	Y	N	N	Y	Y	Y	Y	Y	Y	17	High
Drummond et al. [54]	Y	Y	Y	Y	Y	Y	N	Y	Y	Y	Y	Y	N	N	Y	Y	Y	Y	Y	NR	16	High
Dutta et al. [65]	Y	Y	N	Y	Y	N	N	Y	Y	Y	Y	Y	N	Y	Y	Y	Y	Y	N	Y	17	High
Ho et al. [45]	Y	Y	N	Y	Y	Y	N	Y	Y	Y	Y	Y	N	Y	Y	Y	Y	Y	N	Y	18	High
Matias and Fontaine [57]	Y	Y	N	Y	Y	N	N	Y	Y	Y	Y	Y	N	N	Y	Y	Y	Y	Y	NR	14	Moderate
Matias et al. [66]	Y	Y	N	Y	Y	N	N	Y	Y	Y	Y	Y	N	Y	Y	Y	Y	Y	Y	Y	16	High
Pan et al. [25]	Y	Y	Y	Y	Y	Y	N	Y	Y	Y	Y	Y	N	Y	Y	Y	Y	Y	Y	Y	18	High
Recuero and Segovia [32]	Y	Y	N	Y	Y	Y	N	Y	Y	Y	Y	Y	N	Y	Y	N	Y	Y	N	NR	16	High
Somech and Drach-Zahavy [67]	Y	Y	Y	Y	Y	Y	N	Y	Y	Y	Y	Y	N	N	Y	Y	Y	Y	N	NR	17	High
Kulik [68]	Y	Y	N	Y	Y	N	N	Y	Y	Y	Y	Y	N	Y	Y	Y	Y	Y	N	NR	16	High
Mean																					16.62	
Standard Deviation																					1.12	

Legend: Y = Yes, N = No, NR = Don’t know, * = Items 13 and 19 are reverse scored. Note: The full list of items is reported in the pre-registered document of the present review.

## Data Availability

Not applicable.

## Supplementary Materials

The following supporting information can be downloaded at: https://www.mdpi.com/article/10.3390/ijerph20216992/s1, Document S1: PRISMA checklist.
